# Desmoid Tumor of the Buttock in a Preadolescent Child

**Published:** 2011-03-10

**Authors:** Yogesh Kumar Sarin, Nita Khurana

**Affiliations:** Department of Pediatric Surgery, Maulana Azad Medical College New Delhi, India

**Keywords:** Desmoid Tumor, Aggressive fibromatosis, Musculoaponeurotic fibromatosis, Fibrosarcoma, Recurrence

## Abstract

Extra-abdominal desmoid tumors are circumscribed but non-capsulated neoplasms of differentiated fibrous tissue arising from musculoaponeurotic tissues. They tend to be locally infiltrative, resulting in a high rate of local recurrence without metastasis, following surgical resection. We report a 9-year-old boy who had a large desmoid tumor in his right buttock that was successfully excised.

## INTRODUCTION

Desmoid tumors (aggressive fibromatosis, musculo-aponeurotic fibromatosis) are poorly understood rare tumors of childhood. Enzinger and Weiss had classified desmoid tumors into superficial (palmar, plantar, and penile fibromatoses), and deep [extra-abdominal (head and neck, chest wall, back, extremities), abdominal [(abdominal wall); and intra-abdominal (pelvic, mesenteric)] types [1].

Extra-abdominal desmoid tumors vary from benign nodules to infiltrating masses in their extent. The common sites of occurrence in children are head and neck and the shoulder regions. Hip and buttock in children account for only 8% of all desmoid tumors [2].

We report a successful surgical extirpation of desmoid tumor arising from buttock in a child, and briefly discuss management of this rare tumor in context of the particular anatomic site.

## CASE REPORT

A 9-year-old boy presented with right-sided sciatica and gradually increasing swelling in the right buttock of 6-month duration. His pain was constant but unrelated to movement or activity. There were no significant constitutional symptoms. Examination revealed a mildly tender, firm to hard, fixed, large mass measuring 8.5 X 7.5 cm in the outer upper part of the right buttock. There was no lesion elsewhere in the body.

Chest radiograph was unremarkable. MRI showed a large, relatively well defined mass lesion measuring 5.5 x 7.5 x 8.5 cm in the right gluteal region involving the gluteus maximus, medius and minimus muscles and infiltrating into the pyriformis. The lesion appeared hypointense on T1 -weighted and hyperintense on T2 -weighted images with marked heterogeneity (Fig. 1). The mass was abutting the right iliac blade, though the cortical outline of the bone was well maintained. Fine needle aspiration cytology of the lesion was reported as benign spindle cell lesion with a differential diagnosis of proliferative fasciomyositis, and desmoid tumor.

**Figure:**
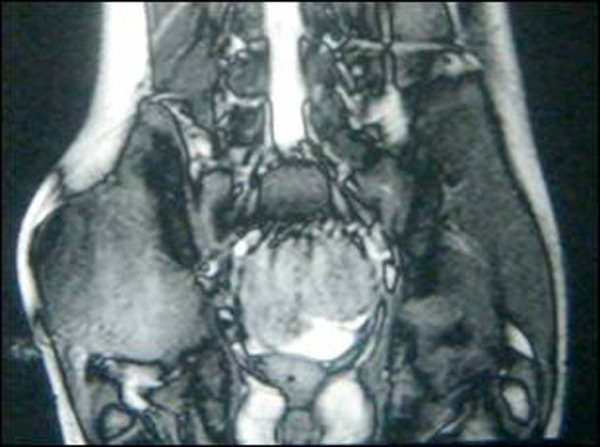
Figure 1: MRI of right gluteal region

A near-total excision of mass was done after an arduous dissection. The excised mass was firm to hard, grey lesion that measured 9 x 7 x 4 cm. It was diagnosed as desmoid tumor on histopathology. Surgical margins of resected specimen were not free of tumor (Fig. 2). The post-operative period was uneventful. No adjuvant therapy was administered. The child is under close follow up for six months and no recurrence is noted so far.

**Figure:**
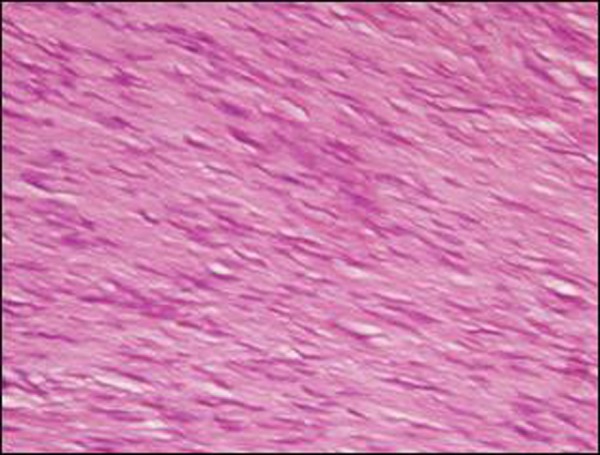
Figure 2: Well differentiated and poorly cellular fibrous tissue with no increased mitotic activity, nuclear pleomorphism or necrosis.

## DISCUSSION

Desmoid tumors are rare benign mesenchymal tumors that may occur in non-syndromic or syndromic forms. The syndromic form is termed as Gardner’s syndrome where there may be multiple desmoid tumors in the soft tissues and the mesentery in association with colonic polyps, sebaceous cyst and ostomas [3].

Radiographic findings in the childhood desmoid tumors are nonspecific, showing appearances from a localized, circumscribed soft tissue mass to an aggressive tumor with local invasion, as seen with malignant neoplasm. The plain film, CT scan, sonographic, and MR characteristics are similar to those of other benign and malignant mesenchymal tumors. Although these examinations are not specific, they can help define tumor margins and extent [4].

The definite diagnosis of desmoid tumors is made histologically only on biopsy or after surgery. Pathological examination reveals a poorly circumscribed, firm, infiltrative grayish-white trabeculated mass that blends into the adjacent soft tissues. Elongated, slender, spindle-shaped cells and dense, often hyalinized, collagen fibers are arranged in bundles. The cellularity of the lesions and mitoses are quite variable but rarely of the degree to suggest a fibrosarcoma. In fact, fibrosarcoma of soft tissues have hardly ever been reported between the age of 6 months and 10 years [5].

The only known treatment in childhood desmoid tumors is wide surgical extirpation. Local recurrences are common with some of the series having reported a recurrence rate of 60%. Positive surgical margins at initial resection predict future recurrence. The anatomic site, size of the mass and histopathologic features are unreliable indicators. Simple excision has been known to have a higher recurrence rate as compared to wide extirpation, but the latter may not be always possible. As such, extensive extirpative surgeries have been known to result in major morbidity. Fortunately, the histologic pattern remains more or less static through a number of local recurrences. It would never become mitotically active and evolve into a fibrosarcoma. An increase in mature collagen may be the only noticeable change [5, 6, 7].

Radiotherapy has been used for local recurrence following one or more surgical resections in adults. Radiotherapy has been also administered for non-resectable desmoid tumors as well as those that could be excised only sub-totally. Post-operative radiation can improve the rate of local control for patients with a high risk of recurrence. As desmoid tumors tend to be locally infiltrative, fields must be very generous to prevent marginal recurrence [8, 9].

Systemic chemotherapy offers an alternative to ablative surgery in the event of local failure following radiation therapy. Combination chemotherapy has also been advocated in unresectable tumors. Regional chemotherapy has also been used in conjunction with wide excision with persistent, widely invasive desmoid tumors. The precise indication for their use in childhood desmoid tumors is not well defined [8, 9, 10].

For adult patients with desmoid tumors that are not amenable to surgery or radiation therapy, the use of hormonal agents and nonsteroidal antiinflammatory drugs (NSAIDs) have been attempted, with some success [10].

Rarely, an expectant therapy has been advocated for unresectable lesions as some of these tumors have been known to regress spontaneously. Spontaneous regression has been noted in female subjects at the onset of either the menarche or the menopause, suggesting that hormonal change might be involved. The premise of such an advocacy is that it is not a malignant condition and has never metastasized. But then there are other researchers who report that the cells of desmoid tumor were highly proliferative with their biological activity bearing resemblance to fibrosarcoma and believe in grouping desmoid tumors as of low grade malignancy [7, 11].

## Footnotes

**Source of Support:** Nil

**Conflict of Interest:** None declared
